# Age-Stratified Analysis of the Clinical Efficacy of Subcutaneous Immunotherapy for Allergic Rhinitis in Chinese Patients

**DOI:** 10.3390/biomedicines13112831

**Published:** 2025-11-20

**Authors:** Ling Jin, Kai Fan, Shican Zhou, Yang Wang, Shiwang Tan, Bojin Long, Shaoqing Yu

**Affiliations:** 1 Department of Otolaryngology, Tongji Hospital, School of Medicine, Tongji University, Shanghai 200065, China; 2 Department of Allergy, Tongji Hospital, School of Medicine, Tongji University, Shanghai 200065, China

**Keywords:** age, efficacy, rhinitis allergic, subcutaneous immunotherapy

## Abstract

**Background/Objectives:** To investigate the relationship between patient age and the clinical efficacy of subcutaneous immunotherapy (SCIT) for allergic rhinitis (AR), aiming to provide a reference for patient selection and efficacy improvement in clinical practice. **Methods:** We conducted a retrospective statistical analysis of clinical data from 240 AR patients who underwent standardized house dust mite (HDM) SCIT for at least 6 months at our hospital between 2019 and 2025. Patients were stratified into four age groups (children, young adults, middle-aged adults, and the elderly) according to the World Health Organization (WHO) classification. The clinical efficacy, nasal symptom scores, Rhinoconjunctivitis Quality of Life Questionnaire (RQLQ) scores, peripheral blood regulatory T cell (Treg) and regulatory B cell (Breg) levels, and adverse reactions were analyzed across these age strata. Additionally, to investigate the underlying mechanisms, we utilized a public single-cell transcriptomic dataset (GSE176269; *n* = 35, age 4 months-65 years) to assess the relationship between T cell senescence and age through data integration and senescence gene set scoring. For multiple comparisons, the significance level was adjusted using the Bonferroni method. This adjustment ensured the overall significance level (α) of the study was maintained at 0.05, and the final adjusted significance level (α′) for each age group was 0.0125. **Results:** The overall response rate for the entire cohort was 62.5%. Age-stratified analysis revealed a significantly higher response rate in children (83.3%) compared to middle-aged and elderly patients (48.5% and 20%, respectively), with the difference being statistically significant (*p* < 0.001). Following treatment, both total nasal symptom scores and RQLQ scores decreased significantly across all age groups compared to baseline (*p* < 0.001). Peripheral blood Treg and Breg levels increased post-treatment in all age groups; however, the increase was not statistically significant in the middle-aged and elderly groups (*p* > 0.0125). The incidence of systemic adverse reactions was 4.17% (all Grade I), occurring primarily in the child and young adult groups, but the difference among age groups was not statistically significant (*p* > 0.0125). Mechanistically, our single-cell analysis revealed that T cells within the nasal mucosa exhibit significant age-dependent senescence. **Conclusions:** SCIT is a safe and effective treatment for AR across all age groups. However, pediatric patients appear to derive greater benefit compared to middle-aged and elderly patients, a finding that corresponds with age-stratified immunological data. Therefore, different efficacy expectations should be considered when selecting SCIT for patients of varying ages, and future research should explore strategies targeting T cell senescence to enhance desensitization efficacy in elderly patients.

## 1. Introduction

Allergic rhinitis (AR) is an allergic disease of the nasal mucosa characterized by symptoms such as nasal itching, sneezing, profuse clear rhinorrhea, and nasal congestion. It can lead to secondary conditions including secretory otitis media, sinusitis, and nasal polyps, and may also trigger or exacerbate asthma, thereby severely impacting patients’ health and quality of life. The global prevalence of AR is estimated at 10–25%, affecting approximately one billion people [1,2], with a continuously rising trend [3,4,5,6,7]. Standard treatments, including antihistamines and intranasal corticosteroids, often provide only temporary symptom relief [8,9] and may be ineffective for a subset of patients [10,11].

Allergen-specific immunotherapy (AIT) is an immunomodulatory process that induces desensitization and immunological tolerance by administering specific allergens [12,13]. It can effectively prevent symptom progression, reduce the risk of sensitization to new allergens [14,15], and lower the likelihood of developing asthma. It is currently the only therapeutic approach capable of modifying the natural course of allergic diseases and achieving long-term efficacy [16,17]. The recommended duration for AIT in AR is 3 to 5 years, with overall efficacy rates ranging from 52.0% to 86.4% [18,19]. While some studies suggest no significant difference in efficacy between children and adults [20,21] and recommend AIT for the elderly with no upper age limit [22,23,24], our clinical observations suggest that the efficacy of AIT in older adults may not be as robust as in younger populations. This prompted us to analyze real-world data from AR patients undergoing AIT to investigate the correlation between age and treatment efficacy.

AIT is mainly administered as subcutaneous immunotherapy (SCIT) or sublingual immunotherapy (SLIT). We focused on SCIT in the present study because of its standardized treatment protocols, systematic management, and the relative ease of obtaining reliable clinical and laboratory data. Previous studies indicate that the efficacy of AIT typically becomes apparent after approximately six months of treatment [25,26]. Therefore, to ensure comparability and adequate sample size, we evaluated short-term efficacy at six months. This study provides an age-stratified analysis of the therapeutic effects of SCIT.

## 2. Materials and Methods

### 2.1. Study Design

**Figure 1:**
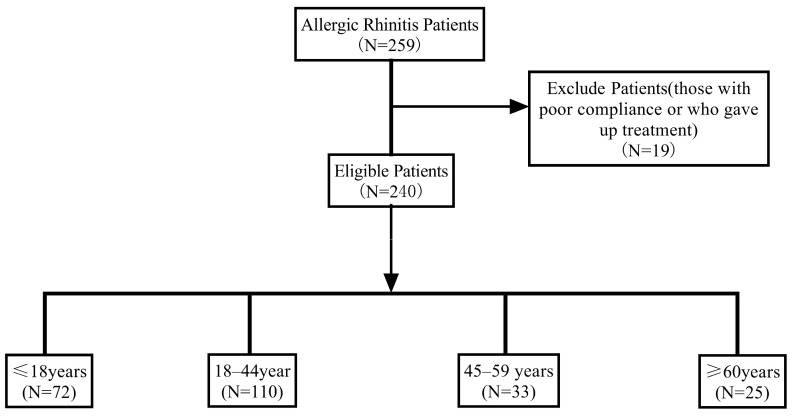
Study flow diagram.

This was a real-world retrospective study. We collected the clinical data of patients with allergic rhinitis (AR) who were diagnosed as house dust mite (HDM)–sensitized based on medical history combined with allergen testing, underwent standardized subcutaneous immunotherapy (SCIT) with HDM extracts at our hospital between July 2019 and March 2025, and completed at least 6 months of treatment. The evaluation endpoint was uniformly set at 6 months after initiation of SCIT. At this time point, changes from baseline in clinical indicators (including nasal symptom scores, quality of life scores, and immunological parameters) were statistically analyzed, with further analyses performed after stratification by age. Because HDM-sensitized AR represents the largest patient population in our region and SCIT with HDM allergen extracts is a well-established and standardized procedure, we selected AR patients undergoing HDM-specific SCIT as the study population (Figure 1). This study was approved by the hospital’s ethics committee (Approval No: K-2019-008).

### 2.2. The Inclusion and Exclusion Criteria of Patients

Because this study was a retrospective analysis, all HDM-sensitized AR patients included had fulfilled the indications for SCIT, had no contraindications, and had provided written informed consent prior to treatment. Indications (inclusion criteria) were: (1) diagnosis of AR according to established diagnostic criteria with confirmed HDM sensitization; (2) age ≥ 5 years; (3) symptoms not adequately controlled by conventional pharmacotherapy and allergen avoidance, or requiring high-dose and/or multiple drugs to achieve control; (4) adverse reactions to pharmacological treatment; (5) willingness to avoid long-term medication use; (6) willingness to prevent the onset of AR or asthma. Contraindications (exclusion criteria) were: (1) uncontrolled or severe asthma [forced expiratory volume in 1 s (FEV1) < 70% of predicted] or irreversible obstructive airway disease; (2) active autoimmune disease; (3) malignancy; (4) current use of β-blockers or angiotensin-converting enzyme inhibitors; (5) severe cardiovascular disease; (6) autoimmune disorders; (7) severe psychiatric illness or poor compliance; (8) primary or secondary immunodeficiency; (9) pregnancy [27,28].

### 2.3. The Diagnosis of AR

Diagnosis of AR: All patients were diagnosed according to guideline criteria, based on typical allergic history, clinical manifestations, and positive allergen testing. (1) Typical symptoms: two or more of paroxysmal sneezing, watery rhinorrhea, nasal itching, and nasal congestion, lasting or accumulating for more than 1 h per day, with or without ocular symptoms such as tearing, itching, and redness. (2) Signs: common findings included pale and edematous nasal mucosa and watery nasal secretions. (3) Allergen testing: at least one allergen tested positive, determined by serum specific IgE (sIgE) measurement and/or skin prick test (SPT) [27,28].

(1) Serum Total IgE and sIgE Measurement: Allergen-specific IgE was detected using the EUROIMMUN immunoblotting system. Serum samples and reagents were loaded into microfluidic cartridges for automated analysis against a panel of 19 common allergens. sIgE levels were graded as follows: Grade 0: <0.35 KU/L; Grade 1: 0.35 to <0.7 KU/L; Grade 2: 0.7 to <3.5 KU/L; Grade 3: 3.5 to <17.5 KU/L; Grade 4: 17.5 to <50 KU/L.
(2) Allergen Skin Prick Test (SPT): SPT was performed using a panel of 18 allergen extracts from ALK (Denmark). Wheal responses were read 15 min after pricking. Results were graded based on the SI value (ratio of the allergen wheal diameter to the histamine wheal diameter). SI = 0 was negative; SI < 0.5 was (+); 0.5 ≤ SI < 1.0 was (++); 1.0 ≤ SI < 2.0 was (+++); SI ≥ 2.0 was (++++).

### 2.4. Pre- and Post-SCIT Examination and Evaluation

(1) Symptom and Clinical Efficacy Assessment: Following established literature [29,30], four key nasal symptoms (sneezing, rhinorrhea, nasal itching, and nasal congestion) were evaluated using a 4-point scale: 0 = no symptoms; 1 = mild (symptoms present but easily tolerated); 2 = moderate (symptoms bothersome but tolerable); 3 = severe (symptoms intolerable, interfering with daily activities and/or sleep). The symptom improvement rate was calculated using the formula: [(Pre-treatment score − Post-treatment score)/Pre-treatment score] × 100%. Efficacy was categorized as: Marked improvement (>66% improvement), Moderate improvement (26–65% improvement), or Ineffective (<25% improvement) [31,32]. The overall response rate was calculated as the percentage of patients achieving marked or moderate improvement (i.e., >25% improvement).
(2) Rhinoconjunctivitis Quality of Life Questionnaire (RQLQ): Patients completed the RQLQ to assess the impact of AR on daily activities, social functions, sleep quality, nasal and eye symptoms, and emotional well-being. Items were rated on a 0–6 scale (the RQLQ uses a 7-point scale, with 0 = no problem to 6 = extremely problematic), with higher scores indicating greater impairment [33,34].
(3) Immunological Parameter Measurement: Peripheral blood was collected before treatment and after 6 months of therapy to assess the treatment’s effect on the immune system. The proportions of Tregs (CD4^+^CD25^+^CD^127low/−^) and Bregs (CD19^+^CD^24hi^CD^38hi^) within peripheral blood mononuclear cells (PBMCs) were analyzed by flow cytometry. Briefly, PBMCs were stained with anti-CD4-PE, anti-CD25-FITC, and anti-CD127-PC5 monoclonal antibodies to identify Tregs, and with anti-CD19-PE, anti-CD24-PC5, and anti-CD38-FITC antibodies to identify Bregs (Beckman Coulter, Brea, CA, USA). Isotype-matched IgGs were used as controls for non-specific fluorescence. Data were acquired on a FACSCalibur flow cytometer (Beckman Coulter, USA) and analyzed using Cell Quest 3.3 software (BD, USA).

### 2.5. SCIT Protocol

All patients received subcutaneous injections of a standardized house dust mite allergen extract (Alutard SQ^®^, ALK, Hørsholm, Denmark). The treatment consisted of two phases. The first was the build-up phase, involving weekly injections of increasing doses from four vials with concentrations of 100, 1000, 10,000, and 100,000 SQ-U/mL. This phase typically lasted 16 weeks (4 months), starting from 0.2 mL of the first vial and escalating to the maximum tolerated dose. The second was the maintenance phase, where the maximum tolerated dose achieved was administered every 2–4 weeks, with the interval gradually extending to 4–8 weeks. The total recommended treatment course is 3–5 years. This analysis included patients who had completed the build-up phase and entered the maintenance phase, with a total treatment duration of at least 6 months. All enrolled patients regularly used triamcinolone acetonide nasal spray at the beginning of treatment, with usage tapered or discontinued as desensitization effects emerged. Apart from occasional use of loratadine for allergic reactions, patients did not take long-term antihistamines.

### 2.6. Observation of Adverse Reactions

All adverse reactions occurring during the 30 min post-injection observation period and thereafter were recorded and assessed for causality by a physician. Systemic reactions were graded according to the European Academy of Allergy and Clinical Immunology (EAACI) systemic reaction grading system [32,33]: Grade 0: No symptoms or non-specific reaction; Grade I: Mild systemic reaction (e.g., localized urticaria, rhinitis, mild asthma with PEF decrease <20% from baseline); Grade II: Moderate systemic reaction (e.g., moderate urticaria, moderate asthma with PEF decrease <40%); Grade III: Severe (non-fatal) systemic reaction (e.g., rapidly developing [<15 min] generalized urticaria, angioedema, or severe asthma with PEF decrease >40%); Grade IV: Anaphylactic shock (e.g., immediate pruritus, flushing, erythema, generalized urticaria, stridor, asthma, hypotension).

### 2.7. Single-Cell Transcriptomic Analysis

To investigate the immunological mechanisms underlying the age-related decline in SCIT efficacy, we conducted a bioinformatic re-analysis of a publicly available single-cell RNA sequencing (scRNA-seq) dataset of human nasal mucosa (GEO accession: GSE176269), comprising 35 healthy donors aged 4 months to 65 years [35].

Raw count matrices were processed in Seurat v4.3.0 (R 4.2.2). Quality control (QC) was performed by excluding cells with <200 or >6000 detected genes and cells with >15% mitochondrial transcripts. Potential doublets were removed using the DoubletFinder (v2.0.3) package. Following normalization and scaling, datasets were integrated and batch effects across donors were corrected using the Harmony (v0.1.0) algorithm.

Cell type annotation was carried out using canonical marker genes reported in the original study and verified with published reference datasets. To quantify cellular senescence, we applied the AddModuleScore function in Seurat with the SenMayo senescence gene set (recently curated and validated for aging studies). For each cell, a senescence score was calculated and compared across age groups.

### 2.8. Statistical Analysis

Patients were stratified into four groups based on the WHO age classification: children (<18 years), young adults (18–44 years), middle-aged adults (45–59 years), and the elderly (≥60 years). Quantitative data are presented as mean ± standard deviation (x ± s), while qualitative data are shown as counts and percentages. Paired t-tests were used to compare quantitative data before and after treatment. Chi-square tests or Fisher’s exact tests were used for qualitative data. When conducting comparisons of quantitative data across multiple groups, analysis of variance (ANOVA) was used, a *p*-value < 0.05 was considered statistically significant. For multiple comparisons, the significance level was adjusted using the Bonferroni method. This adjustment ensured the overall significance level (α) of the study was maintained at 0.05, and the final adjusted significance level (α′) for each age group was 0.0125. The calculation formula is α′ = α/k, where k represents the number of experimental groups. All statistical analyses were performed using SPSS version 26.0.

## 3. Results

### 3.1. Study Cohort

A total of 240 patients (140 males, 100 females) who had completed at least 6 months of SCIT were included. The ages ranged from 5 to 67 years, with a mean age of 31.38 ± 17.629 years. The cohort included patients with AR alone as well as those with comorbid allergic conjunctivitis or mild asthma(Table 1). Co-sensitizations included cat dander (+) (*n* = 1), cat dander (++) *(n* = 2), dog epithelium (++) (*n* = 2), mugwort (+) (*n* = 1), and willow/poplar/elm (+) (*n* = 1).

### 3.2. Efficacy Evaluation by Age Stratification

#### 3.2.1. Response Rate by Age Group

**Figure 2:**
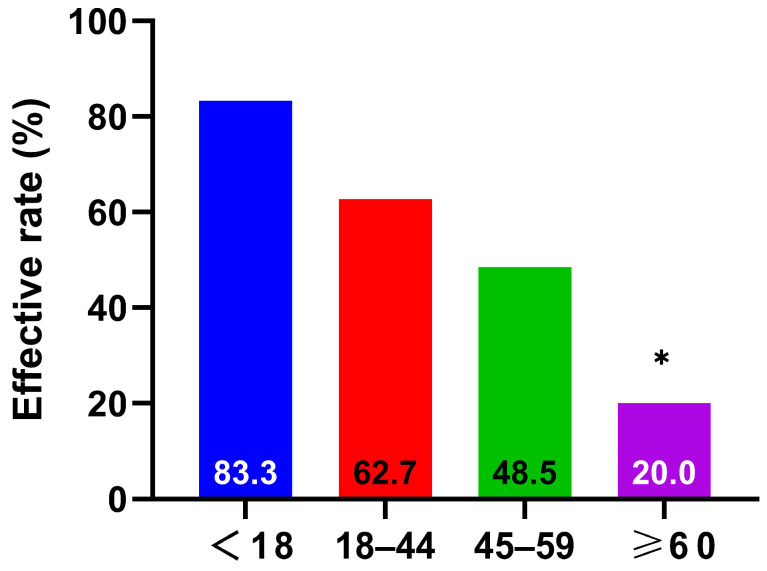
Age-stratified results of the treatment efficacy of SCIT, * *p* < 0.0125. It shows the efficacy rates of four age groups, with the elderly aged over 60 years having the lowest efficacy rate (20%, *p* < 0.0125).

After 6 months of treatment, 150 out of 240 patients showed improvement, comprising 90 with marked improvement and 60 with moderate improvement, yielding an overall response rate of 62.5%. Stratified analysis by age showed response rates of 83.3% for the <18 years group, 62.7% for the 18–44 years group, 48.5% for the 45–59 years group, and 20% for the ≥60 years group. The difference among the groups was statistically significant (*p* < 0.001) (Figure 2).

#### 3.2.2. Nasal Symptom Scores by Age Group

**Figure 3:**
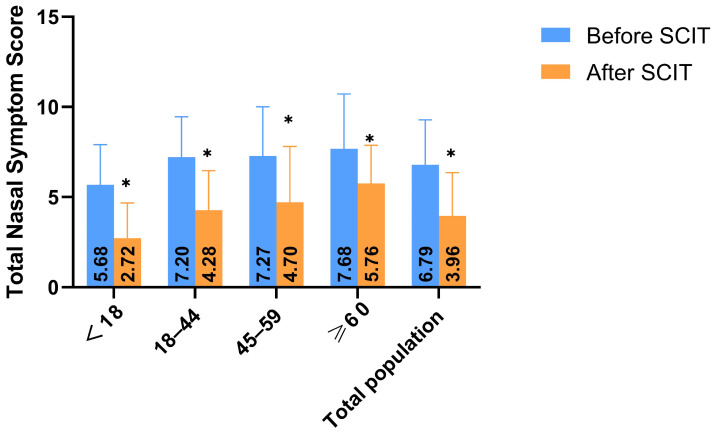
Age-stratified analysis results of the total nasal symptom scores before and after SCIT treatment, * *p* < 0.0125. The total nasal symptom scores of all age groups decreased compared with those before treatment, and the differences were significant (*p* < 0.0125).

**Figure 4:**
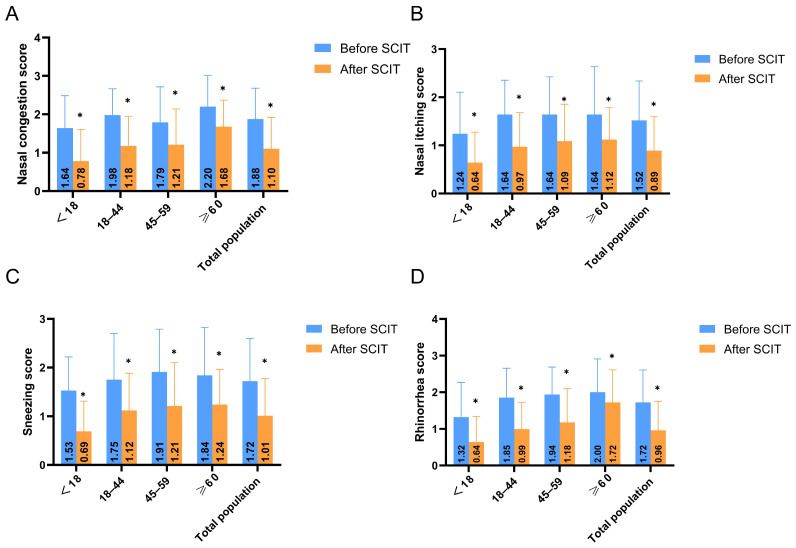
Age-stratified analysis results of four main symptoms (**A**) nasal congestion, (**B**) nasal itching, (**C**) sneezing, and (**D**) clear nasal discharge) before and after SCIT treatment, * *p* < 0.0125. It shows that the four symptoms in all age groups were improved after treatment compared with those before treatment, with significant differences (*p* < 0.0125).

Post-treatment total nasal symptom scores were significantly lower than pre-treatment scores in all age groups (*p* < 0.0125) (Figure 3). Scores for nasal congestion, itching, sneezing, and rhinorrhea were all significantly reduced post-treatment (*p* < 0.0125) (Figure 4A–D).

#### 3.2.3. RQLQ Scores by Age Group

**Figure 5:**
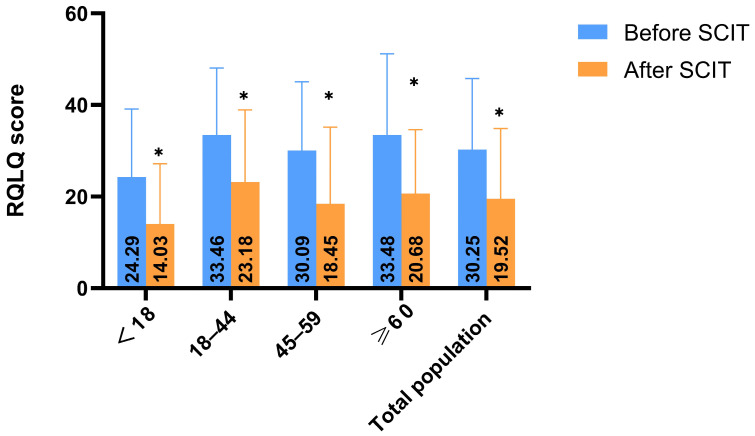
Age-stratified analysis results of RQLQ before and after SCIT treatment, * *p* < 0.0125. The RQLQ scores of all age groups decreased compared with those before treatment, and the differences were significant (*p* < 0.0125).

Post-treatment RQLQ scores were significantly lower than pre-treatment scores in all age groups (*p* < 0.0125) (Figure 5).

#### 3.2.4. Peripheral Blood Treg and Breg Changes by Age Group

**Figure 6:**
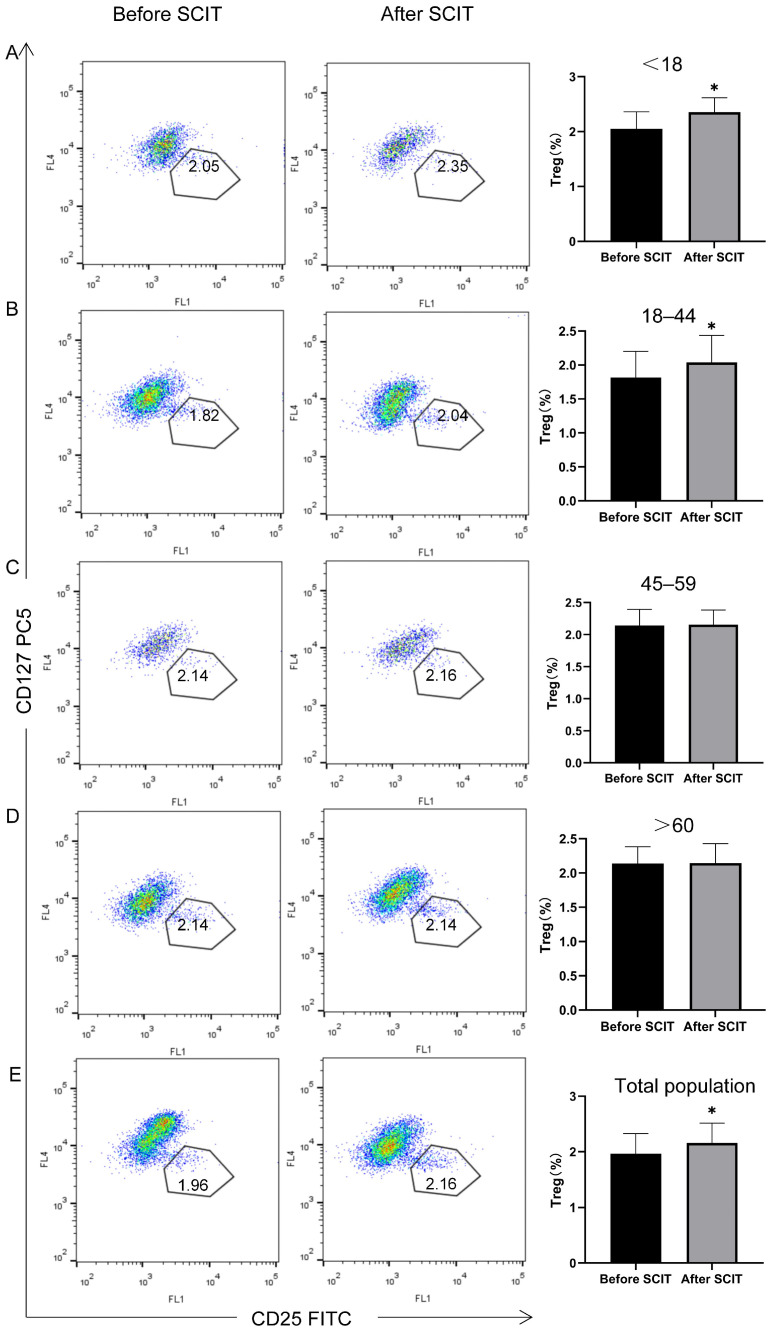
Age-stratified analysis of the flow cytometry detection results of peripheral blood Treg before and after SCIT treatment, * *p* < 0.0125. In the flow scatter plots, different color clusters represent different cell populations, and the target cell population is defined by gating (the circled area in the figure). The left side shows the flow scatter plots before treatment, the middle shows those after treatment, and the right side shows the statistical bar charts of the Treg cell populations in the corresponding age groups ((**A**) <18 years old; (**B**): 18–44 years old; (**C**) 45–50 years old; (**D**) >60 years old)/total population (**E**) before and after treatment. The number of Treg cells in the elderly and middle-aged groups (**C**,**D**) showed little change after treatment (no significant difference).

**Figure 7:**
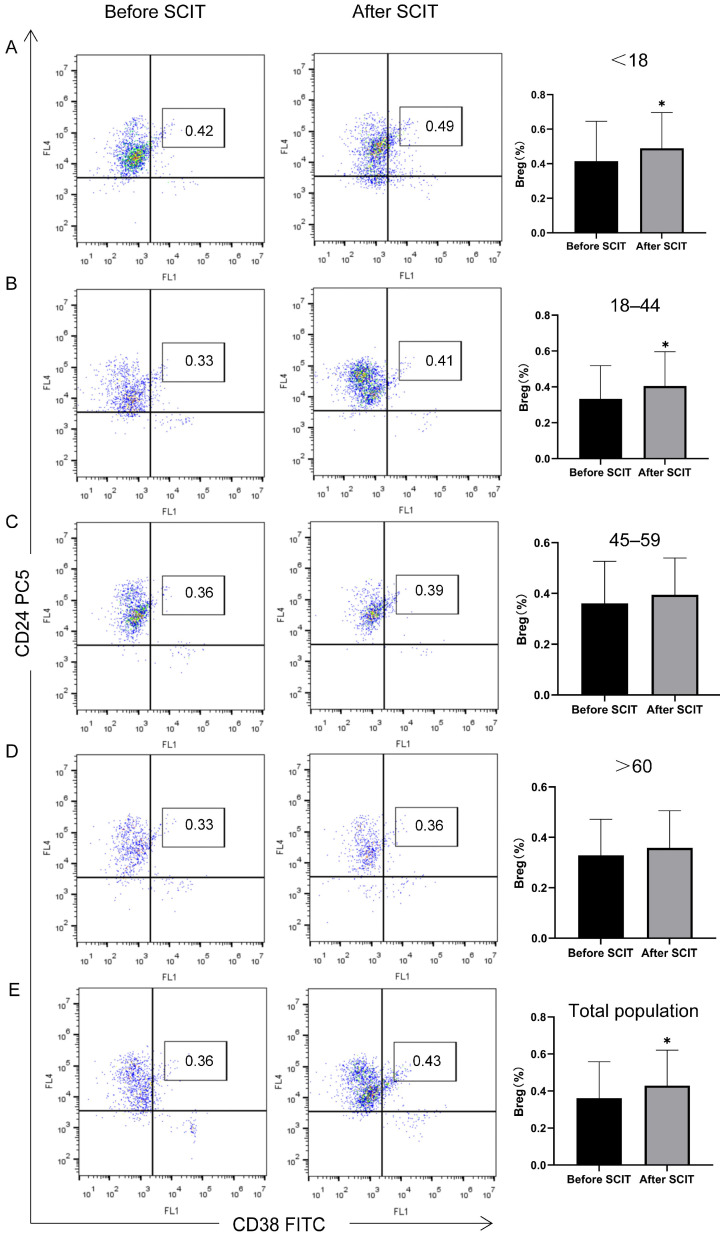
Age-stratified analysis of flow cytometry detection results of peripheral blood Breg before and after SCIT treatment, * *p* < 0.0125. The left side shows the flow scatter plots before treatment, the middle shows those after treatment, and the right side shows the statistical bar charts of Breg cell populations in the corresponding age groups (**A**) <18 years old; (**B**) 18–44 years old; (**C**) 45–50 years old; (**D**) >60 years old)/total population (**E**) before and after treatment. After treatment, the number of peripheral blood Breg cells in the two age groups (**A**,**B**) increased (*p* < 0.0125), while there was no significant difference in the change in Breg cell number in groups (**C**,**D**).

In the age-stratified analysis, post-treatment levels of Tregs and Bregs showed no significant change from baseline in the middle-aged and elderly groups (*p* > 0.0125). In contrast, Treg and Breg levels were significantly increased post-treatment in the younger age groups (* *p* < 0.0125) (Table 2, Figure 6 and Figure 7).

### 3.3. Adverse Reactions by Age Stratification

No severe adverse reactions occurred among the 240 patients. According to the EAACI grading system [36], there were only 5 cases of Grade I systemic adverse reactions and 9 cases of local reactions (pruritus and/or induration), with 3 patients experiencing both. Systemic reactions were more common in females (4 out of 5 cases), as were local reactions (7 out of 9 cases). Although no adverse reactions were recorded in the elderly group, the age-stratified analysis showed no statistically significant difference in the incidence of either systemic or local reactions among the groups (Table 3).

### 3.4. Nasal Mucosal T Cells Exhibit Significant Age-Dependent Senescence

**Figure 8:**
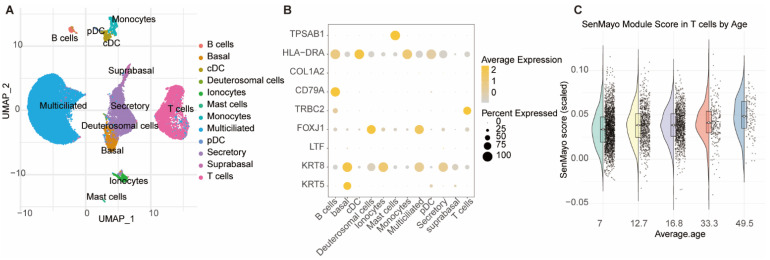
Age-Dependent T Cell Senescence in the Human Nasal Mucosa. (**A**) UMAP plot of 12 distinct cell types identified from the nasal mucosa after Harmony integration. Each point is a single cell, colored by its annotated identity. (**B**) Dot plot showing the expression of key marker genes used for cell type annotation. Dot size indicates the percentage of expressing cells, and color intensity reflects the average expression level. (**C**) Violin plots of T cell senescence scores stratified by donor age group, demonstrating a significant positive correlation between cellular senescence and age.

To investigate a biological basis for the diminished Treg/Breg responses and reduced clinical efficacy observed in older patients, we analyzed the nasal mucosal immune microenvironment using the public scRNA-seq dataset. Initial analysis identified 12 distinct cell types, including T cells, B cells, basal cells, ciliated cells, and mast cells (Figure 8A), which were annotated based on the expression of canonical marker genes (Figure 8B).

Crucially, our targeted analysis revealed that the senescence score of the T cell population was strongly and positively correlated with donor age (Figure 8C). This finding provides direct molecular evidence for progressive, age-dependent immunosenescence within the nasal T cell compartment. This offers a compelling biological rationale for the impaired immune tolerance induction and reduced SCIT efficacy observed in our older AR patient cohort.

## 4. Discussion

In our cohort of 240 AR patients, a comparison of pre- and post-treatment data after 6 months revealed significant decreases in both nasal symptom scores and RQLQ scores (*p* < 0.001), with an overall response rate of 62.5%, consistent with published literature [18,19]. This reaffirms that SCIT is effective for AR, successfully controlling symptoms like rhinorrhea, nasal congestion, itching, and sneezing, and thereby improving patients’ quality of life. The significant improvements in nasal symptom and RQLQ scores within each age group demonstrate that SCIT is effective across all ages, including the elderly. Beyond SCIT, guideline-supported options include intranasal corticosteroids (first-line), oral/intranasal antihistamines, leukotriene receptor antagonists, and fixed-dose steroid–antihistamine sprays, alongside saline irrigation and environmental control. For selected patients, sublingual immunotherapy is an alternative; in refractory cases, short courses of systemic corticosteroids for severe flares and biologics (e.g., anti-IgE) may be considered.

Since nasal symptom scores and RQLQ scores are subjective self-reported measures, there can be variability in scoring among individuals for the same condition, making it difficult to ensure baseline balance across groups. We, therefore, used the response rate for our age-stratified analysis. The calculation of the response rate is based on each patient’s self-assessment against a standardized criterion, which minimizes the influence of confounding factors. Our results (Figure 2) from the age-stratified analysis (children, young adults, middle-aged, elderly) showed response rates of 83.3% (<18 years), 62.7% (18–44 years), 48.5% (45–59 years), and 20% (≥60 years), with a statistically significant difference (*p* < 0.001). This suggests that the efficacy of SCIT is comparatively lower in older adults.

For over a century, SCIT has been used to treat AR. Most studies on the relationship between SCIT efficacy and age have focused on comparing children and adults, often reporting no significant difference [20,21]. Theoretically, because the Th2 memory response to allergens is not fully established in childhood, immunotherapy initiated at this stage is more likely to effectively down-regulate the Th2 response. Indeed, some studies have found that immunotherapy is more effective in children and young adults than in older adults [37,38,39,40], which aligns with the results of our age-stratified efficacy analysis.

Immunosenescence is a critical aspect of the aging process, leading to numerous changes in the immune system. The reduced efficacy of SCIT in the elderly population observed in our study is likely associated with immunosenescence. To explore this, we also conducted an age-stratified analysis of changes in relevant immunological markers (peripheral blood Tregs and Bregs) before and after treatment. Notably, our bioinformatic analysis of the public nasal mucosa scRNA-seq dataset (GSE176269) directly supports this hypothesis. The finding that the senescence module score in local T cells is significantly correlated with age provides direct evidence of an age-dependent senescence phenomenon within the nasal immune microenvironment. The finding that the senescence module score in local T cells is significantly correlated with age provides direct evidence of an age-dependent senescence phenomenon within the nasal immune microenvironment, consistent with prior reports of airway immunosenescence; however, these exploratory findings from healthy donors do not constitute mechanistic evidence for our SCIT cohort. This molecular evidence aligns perfectly with our clinical observations of insufficient Treg/Breg upregulation and diminished SCIT efficacy in older patients. It reinforces the conclusion that immunosenescence is a key mechanism impacting desensitization outcomes. Therefore, future strategies aimed at intervening in or reversing T cell functional decline may represent a promising approach to enhance SCIT efficacy in middle-aged and elderly AR patients.

The induction and maintenance of immunological tolerance are crucial mechanisms for maintaining immune homeostasis and preventing allergic inflammation [41]. AIT primarily restores impaired allergic tolerance by inducing the generation and activation of Treg and Breg cells [42]. Treg cells are key mediators of immune tolerance and homeostasis [43]. They prevent and suppress allergic inflammation by inhibiting the degranulation of mast cells and basophils and by negatively regulating the function of eosinophils, dendritic cells, and T and B lymphocytes [44]. A study by Kouzegaran et al. found that the immune mechanisms underlying symptom relief and improvement in AR patients undergoing SCIT included an increase in the number of Treg cells and elevated levels of TGF-β and IL-10 [45]. Recent research has shown that Breg cells regulate immune responses by secreting inhibitory cytokines (IL-10, IL-35, and TGF-β) [46,47], which can down-regulate inflammatory responses and induce tolerance [48]. Bregs can produce IL-10 and TGF-β to promote IgG4 synthesis, thereby inhibiting the activity of mast cells and basophils. They can also suppress Th2-mediated inflammation, either directly or by promoting Treg generation [47,48,49], leading to the amelioration of AR symptoms and the establishment of immune tolerance [50].

Consequently, monitoring Treg and Breg levels has been proposed as a potential method for evaluating or predicting the efficacy of SCIT for allergic rhinitis [51,52,53]. Our age-stratified analysis of these immune markers revealed that while Treg and Breg levels increased post-treatment in the middle-aged and elderly groups, the change was not statistically significant. In contrast, the increase was significant in the younger age groups. This suggests that Treg and Breg levels can reflect SCIT efficacy and that the poorer response in older adults may be linked to a decline in the capacity to generate immune tolerance due to immunosenescence.

According to the European Academy of Allergy and Clinical Immunology (EAACI) guidelines [54], adverse reactions to SCIT are classified as local or systemic. Local reactions, such as redness, itching, or swelling at the injection site, are most common Systemic reactions(SR) are typically mild (Grade I or II) [55,56]. The incidence of SR in conventional SCIT is reported to be 0.1–0.2% per injection and 2–5% per patient [57]. In our study, the incidence of SR was 4.17%, all of which were Grade I, consistent with the literature. Age-stratified analysis showed no statistically significant difference among groups, although more cases were observed in the child and young adult groups, indicating a high overall safety profile for SCIT.

A retrospective study by Lim et al. [58] comparing SRs in children and adults undergoing SCIT found a significantly higher overall SR rate in children. Other studies have also reported a higher incidence of SRs in pediatric patients compared to adults [59,60], possibly due to a more active immune system in children. The SRs in our study also occurred mainly in the younger age groups. The lack of statistical significance compared to the elderly group may be due to the small number of cases.

An interesting observation was that adverse reactions, both systemic and local, were more frequent in females (local: 7/9; systemic: 4/5). This finding is consistent with other studies [59,60,61], including a large-scale (*n* = 2200, 30-year) retrospective study from Italy which reported that 70% of patients with adverse reactions were female [61]. This has been speculatively linked to sex hormones, but further mechanistic research is lacking.

The limitations of this study include its single-center, retrospective design. Due to the small sample size and high dropout rate, a long-term efficacy analysis was not performed. Future studies with larger cohorts could allow for a more detailed age-stratified analysis, potentially yielding more specific and valuable results. There are potential confounding factors in this study, such as baseline severity, comorbidities, medication use, compliance, smoking, and seasonal variations that were not fully controlled. Therefore, future multicenter randomized controlled trials are needed to address these confounding factors and validate our findings. Finally, the associations we report between attenuated Treg/Breg responses, higher senescence-associated gene expression and poorer clinical efficacy are correlational; prospective mechanistic studies are needed to test whether these immune changes causally mediate SCIT nonresponse.

## 5. Conclusions

This study, analyzed from a unique age-stratified perspective, confirms that SCIT is an effective and safe treatment for AR. The improvement in allergic symptoms is accompanied by an increase in serum Treg and Breg levels, suggesting that these cells may serve as objective biomarkers for evaluating the clinical efficacy of immunotherapy. Furthermore, the age-stratified analysis of both clinical efficacy and immunological markers revealed a poorer response in middle-aged and elderly patients, a clinical finding supported by objective immunological data. The goal of our age-stratified analysis was to clarify whether differences in clinical efficacy of SCIT are associated with patient age and corresponding immunological changes. By integrating clinical outcomes with laboratory and single-cell data, we aimed to identify objective markers that may explain reduced responses in older patients. This approach provides a framework for improving efficacy assessment and guiding the rational selection of candidates for immunotherapy, particularly in the context of an aging population.

## Figures and Tables

**Table 1:** Demographic baseline information.

	≤18 Years (*n* = 72)	18–44 Year *(n* = 110)	45–59 Years (*n* = 33)	≥60 Years (*n* = 25)	Total (*n* = 240)
Age	11.25 ± 3.368	31.47 ± 7.342	50.73 ± 2.820	63.44 ± 2.200	31.38 ± 17.629
Sex					
Male	45 (62.5%)	61 (55.5%)	18 (54.5%)	16 (64.0%)	140 (58.3%)
Female	27 (37.5%)	49 (44.5%)	15 (45.5%)	9 (36.0%)	100 (41.7%)
Medical history (non-study disease)					
Yes	9 (12.5%)	73 (66.4%)	23 (69.7%)	23 (92.0%%)	128 (53.3%)
No	63 (87.5)	37 (33.6%)	10 (30.3%)	2 (8.0%)	112 (46.7%)
TNSS	5.72 ± 2321	7.22 ± 2.272	7.27 ± 2.730	7.68 ± 3.038	6.83 ± 2.534
RQLQ	24.29 ± 14.815	33.46 ± 14.638	30.09 ± 14.9999	33.48 ± 17.676	30.25 ± 15.523

**Table 2:** Age-stratified analysis results of Treg and Breg detection in peripheral blood before and after SCIT.

	≤18 Years (*n* = 72)	18–44 Year (*n* = 110)	45–59 Years (*n* = 33)	≥60 Years (*n* = 25)	Total (*n* = 240)
Treg (%)					
Before SCIT	2.05 ± 0.31	1.82 ± 0.39	2.14 ± 0.25	2.14 ± 0.25	1.96 ± 0.36
After SCIT	2.35 ± 0.27 *	2.04 ± 0.40 *	2.16 ± 0.23	2.14 ± 0.28	2.16 ± 0.36 *
Breg (%)					
Before SCIT	0.42 ± 0.23	0.33 ± 0.18	0.36 ± 0.17	0.33 ± 0.14	0.36 ± 0.20
After SCIT	0.49 ± 0.21 *	0.41 ± 0.19 *	0.39 ± 0.15	0.36 ± 0.15	0.43 ± 0.19 *

**Table 3:** Age-stratified analysis results of adverse reactions in SCIT. treatment.

	≤18 Years (*n* = 72)	18–44 Years (*n* = 110)	45–59 Years (*n* = 33)	≥60 Years (*n* = 25)	Total (*n* = 240)	*p*
Systemic reactions	2 (5.13%)	3 (4.76%)	0 (0%)	0 (0%)	5 (4.17%)	0.818
Local reactions	5 (12.82%)	3 (4.76%)	1 (9.09%)	0 (0%)	9 (7.5%)	0.340

## Data Availability

The data presented in this study are available on request from the corresponding author due to privacy and ethical reasons.

## Abbreviations

The following abbreviations are used in this manuscript:

SCIT	Subcutaneous immunotherapy.
AR	Allergic rhinitis.
HDM	House dust mite.
WHO	World Health Organization.
RQLQ	Rhinoconjunctivitis Quality of Life Questionnaire.
AIT	Allergen-specific immunotherapy.
SLIT	Sublingual immunotherapy.
SPT	Skin prick tests.
PBMCs	Peripheral blood mononuclear cells.
Treg	regulatory T cell(s).
Breg	regulatory B cell(s).
sIgE	specific immunoglobulin E.
CI	confidence interval.
